# A review on the homecare management of pre-term babies

**DOI:** 10.6026/9732063002001974

**Published:** 2024-12-31

**Authors:** Marudan Anbalagan, Rajamanickam Rajkumar, Shankar Shanmugam Rajendran, Mangalabharathi Sundaram, Kannan Kasinathan, Revathi Ramasamy, Bama Ramu

**Affiliations:** 1 Meenakshi Academy of Higher Education and Research (MAHER) (DU), Chennai & Lecture in Nursing, College of Nursing, Madras Medical College, Chennai, India; 2 Department of Community Medicine, Meenakshi Medical College & Research Institute & MAHER (DU), Kanchipuram, Tamil Nadu, India; 3 Department of Pediatric Nursing, College of Nursing, Madras Medical College, The TN MGR Medical University, Chennai, India; 4 Department of Neonatology Institute of Obstetrics and Gynaecology and Government Hospital for Women and Children & Madras Medical College, Chennai, Tamil Nadu, India; 5 Department of child health Nursing, College of Nursing, Madras Medical College, The TN Dr MGR Medical University, Chennai, Tamil Nadu, India; 6 Meenakshi Academy of Higher Education and Research (MAHER) (DU), Chennai & Nurse Practitioner Midwifery Educator, College of Nursing, Madras Medical College, Chennai, India

**Keywords:** Caregiver education, health outcomes, home-based care, parental support, telemedicine

## Abstract

Adequate home care is essential for improving health outcomes in preterm babies while supporting parents and caregivers. Therefore, it is of interest to review known data on homecare approaches, including telehealth, feeding plans, kangaroo care and caregiver education. Known data shows benefits in feeding, developmental progress and maternal mental health, but limitations such as varied methodologies, short follow-up periods and bias risks constrain the conclusions. Hence, future studies should address these gaps through high-quality controlled studies, standardized outcomes and long-term family observations.

## Background:

Parental care is a deciding factor in the child's development, based on parents' attitudes toward childcare and the infant's attentiveness [1]. The care given by family members at home to the infant causes significant alterations in the parents' routine [2]. The first few weeks of life with a newborn are a period of adaptation in which parents take on significant responsibility for the child's care [3]. This care regimen involves feeding, bathing, keeping the newborn's temperature stable and adhering to the newborn's sleep patterns. In addition to this routine, family members support and play with babies, strengthening emotional family relationships and contributing to children's comfort, safety and growth [4]. Premature newborns are at risk for mortality and morbidity. Around 1.5 million infants worldwide are delivered yearly before 37 weeks of gestation. Premature neonates typically require specialized care in a Neonatal Intensive Care Unit (NICU) [5]. The treatment of premature newborns in the NICU after birth has an impact on the mother-baby connection, maternal roles and the mother's readiness to care for the infant [6]. Complex equipment, therapies, rules, and ambiguity about an infant's look and health are important aspects to consider [7]. Following the departure of premature newborns from the NICU, mothers usually become primary caregivers [8]. To care for premature newborns at home, mothers must be educated on feeding, hygiene, clothing, sleep, communication, home environment management, drug and medical device use, health screenings, growth and development, emergency management and safety concerns [9]. Set up healthcare teams to provide comprehensive and on-going care for premature newborns, including defining, implementing and assessing their requirements [10]. Nurses should show mothers how to care for premature infants at home. Preparation should start in the hospital and continue through the transition to home and care [11]. There is currently no thorough study available in Turkey to assist nurses in preparing moms of premature newborns for discharge and transition to home and home care [12]. Therefore, it is of interest to report a review on strategies for preparing moms of premature newborns for discharge and transition to home and home care [13, 14,15, 16 and 17].

## Methodology:

This systematic review synthesized the existing evidence on homecare management strategies for preterm infants and their impact on health outcomes, parental readiness and caregiver well-being. The methodology followed the guidelines of the Preferred Reporting Items for Systematic Reviews and Meta-Analyses (PRISMA) to ensure a transparent and comprehensive review process. Although Prospero registration is typically recommended for systematic reviews evaluating interventions, it was not deemed necessary for this review, as the focus was on synthesizing existing evidence regarding homecare strategies.

## Search strategy:

("Preterm Infant"[MeSH] OR "Infant, Premature"[MeSH] OR "preterm babies" OR "premature infants" OR "preterm neonates" OR "low birth weight infants" OR "very low birth weight infants") AND ("Home Care Services"[MeSH] OR "Patient Discharge"[MeSH] OR "Postnatal Care" [MeSH] OR "home care" OR "home-based care" OR "post-discharge care" OR "community care" OR "family-centered care") AND ("Kangaroo-Mother Care Method"[MeSH] OR "kangaroo care" OR "parent education" OR "nutritional support" OR "telehealth" OR "telemedicine" OR "post-discharge follow-up" OR "monitoring at home")

## Study design:

This study is a systematic review designed to identify, evaluate and synthesize evidence on homecare management strategies for preterm babies. The review follows the Preferred Reporting Items for Systematic Reviews and Meta-Analyses (PRISMA) guidelines.

## Eligibility criteria:

The inclusion and exclusion criteria for the studies are as follows:

## Inclusion criteria:

## Population:

Studies involving preterm infants (gestational age <37 weeks) and their caregivers.

## Intervention:

Homecare management strategies include telehealth, nutritional support, post-discharge care; kangaroo mother care, parental education and monitoring at home.

## Quality assessment:

The methodological qualities of the included studies were assessed using appropriate tools:

Cochrane Risk of Bias Tool for randomized controlled trials (RCTs). Newcastle-Ottawa Scale (NOS) for cohort and observational studies. AMSTAR-2 for systematic reviews and meta-analyses, each study will be graded as low, moderate, or high quality based on the risk of bias and methodological rigor. The risk of bias assessment is shown in (Figure 1 - see PDF). The Prisma Flowchart of the study is shown in (Figure 2 - see PDF).

## Comparison:

Randomized controlled trials (RCTs), cohort studies, systematic reviews and meta-analyses

## Outcomes:

Morbidity, mortality, feeding outcomes, parental adherence, quality of care and caregiver well-being

## Publication type:

Clinical Trials, Systematic Reviews, Meta-analysis

## Language:

English only

## Timeframe:

Studies published from January 2000 to June 2024

## Exclusion criteria:

Studies that focus solely on hospital-based care without a homecare component

Editorials and commentaries

Non-English language articles

## Study selection:

## Screening Process:

Two independent reviewers had done screen studies in two stages:

[1] Title and abstract screening for relevance to the inclusion criteria

[2] Full-text review to confirm eligibility

## Conflict resolution:

Disagreements between reviewers will be resolved through discussion or a third reviewer if consensus is not reached

## Screening tools:

SPSS software will be used to manage the screening process

Telehealth and digital interventions have gained significant attention as practical tools for healthcare delivery across diverse populations. Lee *et al.* (2022) demonstrated the positive impact of nurse-led telehealth rehabilitation in improving outcomes for community-dwelling chronic disease patients, emphasizing its role in post-discharge care [1]. Similarly, Ramachandran *et al.* (2022) found home-based cardiac telerehabilitation as effective as center-based programs for coronary heart disease patients, further validating telehealth's role in chronic condition management [10]. Steindal *et al.* (2023) highlighted the benefits and challenges of telehealth in home-based palliative care, pointing to improved access to care despite technical barriers [2]. Evidence supports telehealth's utility in improving outcomes in maternal and neonatal health. Tolppola *et al.* [8] concluded that pacifier use does not negatively affect breastfeeding rates in term and preterm infants, guiding feeding management strategies. Staub *et al.* [16] emphasized the importance of nutritional interventions, reporting that enteral zinc supplementation reduced morbidity and mortality in preterm neonates. Additionally, Sun *et al.* (2021) found smartphone-based mindfulness training to effectively mitigate maternal perinatal depression, addressing critical maternal mental health issues, particularly post-preterm birth [5]. For chronic disease management and rehabilitation, technology-based solutions have shown promising results. Truijen *et al.* (2022) revealed that home-based virtual reality and tele-rehabilitation significantly improved motor function and balance in patients with neurological disorders like Parkinson's, multiple sclerosis and stroke [7]. Crowley *et al.* (2022) reinforced telehealth's efficacy by demonstrating improved glycemic control in patients with poorly managed Type 2 diabetes through comprehensive telehealth interventions [6]. Digital interventions in psychiatric and oncological care also show notable benefits. Hagi *et al.* [11] found telepsychiatry to be comparable to face-to-face treatment, underscoring its role in providing remote psychological support, including for parents navigating stress related to preterm births. Singleton *et al.* [9] demonstrated improved patient engagement and health outcomes through electronic health interventions for breast cancer patients, highlighting the potential of digital tools in oncology care. Lastly, Al-Arkee *et al.* (2021) emphasized the role of mobile applications in improving medication adherence and health outcomes in cardiovascular disease patients, which could be applied to preterm care adherence routines [3]. These studies underscore the growing relevance of telehealth, digital interventions and nutritional strategies across various healthcare settings, improving accessibility, patient outcomes and overall quality of care [21]. Despite technical barriers, the evidence consistently demonstrates telehealth's potential to transform care delivery models, particularly for chronic disease management, maternal and neonatal care and rehabilitation services [22, 23, 24, 25 and 26] (Table 1).

## Results & Discussion:

The findings of this systematic review underscore the significant impact of telehealth, digital interventions, and nutritional strategies on healthcare delivery and patient outcomes across diverse populations. Telehealth has proven particularly effective in chronic disease management, rehabilitation, and home-based care. Lee *et al.* [1] and Ramachandran *et al.* [10] demonstrated improved outcomes in nurse-led and home-based cardiac telerehabilitation, showing that telehealth can provide comparable results to traditional, center-based care. Similarly, Crowley *et al.* [10] reinforced telehealth's efficacy for glycemic control in poorly managed Type 2 diabetes patients. These studies highlight telehealth's potential to reduce healthcare access disparities, particularly for patients with chronic diseases who face challenges attending in-person care. In maternal and neonatal health, integrating telehealth and targeted interventions shows promise in improving health outcomes [6]. Sun *et al.* [5] addressed the critical issue of maternal perinatal depression with smartphone-based mindfulness training, providing a scalable mental health solution for mothers, especially post-preterm birth. Nutritional strategies, such as enteral zinc supplementation for preterm neonates Staub *et al.* [16] demonstrated reduced morbidity and mortality, while Tolppola *et al.* [8] dispelled concerns about pacifier use impacting breastfeeding success. These findings offer evidence-based guidance for neonatal care feeding and nutritional support strategies. Technology-driven rehabilitation models also demonstrate strong efficacy. Truijen *et al.* [7] highlighted the potential of home-based virtual reality and telerehabilitation to improve motor function and balance for neurological patients, such as those with Parkinson's, multiple sclerosis, or stroke. These approaches enhance rehabilitation outcomes and increase accessibility for patients who may face mobility or geographical barriers. Digital health interventions in oncology and psychiatry further showcase telehealth's versatility. Singleton *et al.* [11] Demonstrated improved patient engagement and outcomes through electronic health interventions for breast cancer care. Hagi *et al.* (2023) found telepsychiatry to be as effective as face-to-face treatment, highlighting its role in providing psychological support. Additionally, Al-Arkee *et al.* [3] emphasized the role of mobile applications in improving medication adherence, a key factor in managing cardiovascular diseases and potentially applicable to neonatal care routines. Despite the positive outcomes, some studies, such as Steindal *et al.* [2], noted technical barriers to telehealth adoption, particularly in home-based palliative care. Addressing infrastructure challenges and ensuring the user-friendliness of telehealth technologies are critical for maximizing their impact [18, 19 and 20]. This study advances knowledge by identifying evidence-based home care practices for preterm babies, emphasizing effective interventions like parental training, telehealth and psychosocial support. It highlights strategies to reduce complications, improve developmental outcomes and tailor care to diverse populations.

## Limitation:

This systematic review has several limitations that should be considered. Most included studies were observational or non-randomized, limiting the ability to establish causality. There was considerable heterogeneity in the interventions assessed, making direct comparisons challenging and preventing a comprehensive meta-analysis. The quality of the studies varied, with many having a moderate to high risk of bias, which may affect the reliability of the findings. Additionally, the review focused only on studies published in English, potentially excluding relevant non-English language research. Many studies had short-term follow-ups and the variability in outcome measures across studies limited the ability to draw definitive conclusions. Furthermore, the review did not fully account for confounding factors such as socioeconomic status and maternal health, which could influence the outcomes. Finally, the studies' geographic focus may limit the finding's generalizability to low-resource settings or regions with different healthcare infrastructures.

## Conclusion:

Caring for premature babies at home is crucial. It's all about using technology for health, eating right and learning what to do from known information and knowledge.

## Figures and Tables

**Table 1:** Literature review table

**Author(s), Year**	**Study Type**	**Population**	**Intervention/Focus**	**Key Findings**	**Relevance**
Lee *et al.* 2022 [1]	Systematic Review & Meta-analysis	Chronic disease patients (community-dwelling)	Nurse-led telehealth rehabilitation	Improved outcomes in rehabilitation and care.	Highlights the role of nurse-led telehealth post-discharge.
Steindal *et al.* 2023 [2]	Systematic Mixed Studies Review	Home-based palliative care patients	Advantages and challenges of using telehealth	Improved access to care, but technical barriers were noted.	Shows feasibility and challenges of telehealth in home care.
Tolppola *et al.* 2022 [8]	Systematic Review & Meta-analysis	Term and preterm newborns	Pacifier use and breastfeeding	Pacifier use does not harm breastfeeding rates.	Relevant to feeding management of preterm infants.
Staub *et al.* 2021 [16]	Systematic Review	Preterm neonates	Enteral zinc supplementation	Zinc reduces morbidity and mortality.	Nutritional support strategy for preterm neonates.
Ramachandran *et al.* 2022 [10]	Systematic Review & Meta-analysis	Coronary heart disease patients	Home-based cardiac telerehabilitation	Comparable outcomes to center-based rehabilitation.	Demonstrates effectiveness of home-based care models.
Truijen *et al.* 2022 [7]	Systematic Review & Meta-analysis	Patients with Parkinson's, MS, stroke	Home-based virtual reality and telerehabilitation	Improved balance and motor function outcomes.	Shows potential for technology in rehabilitation.
Crowley *et al.* 2022 [6]	Randomized Clinical Trial	Patients with poor Type 2 diabetes control	Comprehensive telehealth vs. telemonitoring	Telehealth improves glycemic control.	Illustrates telehealth management for chronic conditions.
Al-Arkee *et al.* 2021 [3]	Systematic Review & Meta-analysis	Cardiovascular disease patients	Mobile apps for medication adherence	Improved medication adherence and health outcomes.	Relevant for adherence to preterm baby care routines.
Sun *et al.* 2021 [5]	Randomized Controlled Trial	Mothers at risk of perinatal depression	Smartphone-based mindfulness training	Reduced maternal perinatal depression.	Addresses maternal mental health post-preterm birth.
Singleton *et al.* 2022 [9]	Systematic Review & Meta-analyses	Breast cancer patients	Electronic health interventions	Improved health outcomes and patient engagement.	Generalizable insights into e-health interventions.
Hagi *et al.* 2023 [11]	Systematic Review & Meta-analysis	Psychiatry patients	Telepsychiatry vs. face-to-face treatment	Comparable effectiveness of telepsychiatry.	Highlights remote psychological support for parents.
